# Chitosan Nanoparticles Functionalized Viscose Fabrics as Potentially Durable Antibacterial Medical Textiles

**DOI:** 10.3390/ma14133762

**Published:** 2021-07-05

**Authors:** Matea Korica, Zdenka Peršin, Lidija Fras Zemljič, Katarina Mihajlovski, Biljana Dojčinović, Snežana Trifunović, Alenka Vesel, Tanja Nikolić, Mirjana M. Kostić

**Affiliations:** 1 Innovation Center of Faculty of Technology and Metallurgy, University of Belgrade, 11000 Belgrade, Serbia; mkorica@tmf.bg.ac.rs; 2 Institute of Engineering Materials and Design, Faculty of Mechanical Engineering, University of Maribor, 2000 Maribor, Slovenia; zdenka.persin@um.si (Z.P.); lidija.fras@um.si (L.F.Z.); 3 Faculty of Technology and Metallurgy, University of Belgrade, 11000 Belgrade, Serbia; kmihajlovski@tmf.bg.ac.rs (K.M.); tanjanikol@gmail.com (T.N.); 4 Institute of Chemistry, Technology and Metallurgy, University of Belgrade, 11000 Belgrade, Serbia; bmatic@chem.bg.ac.rs; 5 Faculty of Chemistry, University of Belgrade, 11000 Belgrade, Serbia; snezanat@chem.bg.ac.rs; 6 Department of Surface Engineering, Jožef Stefan Institute, University of Ljubljana, 1000 Ljubljana, Slovenia; alenka.vesel@guest.arnes.si

**Keywords:** viscose fabric, TEMPO-oxidation, TEMPO-oxidized cellulose nanofibrils, chitosan nanoparticles, chitosan nanoparticles with embedded zinc ions, washing durable antibacterial textile

## Abstract

This research proposed two pretreatments of viscose fabrics: oxidation with 2,2,6,6-tetramethylpiperidine-1-oxy radical (TEMPO) and coating with TEMPO-oxidized cellulose nanofibrils (TOCN), to introduce functional groups (COOH and CHO) suitable for irreversible binding of chitosan nanoparticles without and with embedded zinc (NCS and NCS + Zn, respectively) and consequently achieving washing durable antibacterial properties of the chitosan nanoparticles functionalized fabrics. The characterizations of pretreated and chitosan nanoparticles functionalized fabrics were performed by FTIR and XPS spectroscopy, elemental analysis, inductively coupled plasma optical emission spectrometry, zeta potential measurements, scanning electron microscopy, determination of COOH and CHO groups content, and antimicrobial activity under dynamic contact conditions. Influence of pretreatments on NCS and NCS + Zn adsorption, chemical, electrokinetic, and antibacterial properties as well as morphology, and washing durability of NCS and NCS + Zn functionalized fabrics were studied and compared. Washing durability was evaluated through changes in the chitosan and zinc content, zeta potential, and antibacterial activity after 1, 3, and 5 washing cycles. Pretreatments improved washing durability of antibacterial properties of chitosan nanoparticles functionalized fabrics. The NCS and NCS + Zn functionalized pretreated fabrics preserved antibacterial activity against *S. aureus* after five washing cycles, while antibacterial activity against *E. coli* was preserved only after one washing cycle in the case NCS + Zn functionalized pretreated viscose fabrics.

## 1. Introduction

Textile materials have great practical value in the medical field and comprise a group of products with ample variations in terms of product performance and values. Viscose is traditionally used as medical textiles for first aid and many clinical and hygienic purposes, primarily due to its biocompatibility [1]. However, the molecular structure of viscose offers numerous possibilities for the development of a broad spectrum of medical textiles with special properties such as, for example, antibacterial [2,3,4,5]. Commonly used compounds for antibacterial functionalization of medical textiles are antibiotics such as quinolones [6], tetracyclines [7], aminoglycosides [8], and cephalosporins [9], which can successfully prevent bacterial proliferation by alteration of protein and nucleic acid syntheses leading to metabolic imbalances and/or the disruption of bacterial cell wall integrity; thus, also inhibiting cell division [10]. However, the use of antibiotics for the antibacterial functionalization of medical textiles is not recommended due to the increasing development of antimicrobial tolerance and resistance [11]. To prevent antibiotic resistance, uncommon, non-antibiotic antibacterial agents, such as chitosan and metals, are included in novel strategies for the development of a novel generation of antibacterial medical textiles based on viscose [5,12,13,14,15,16].

Besides antibacterial, chitosan has many special properties such as biocompatibility [17], biodegradability [18], low toxicity [19], high humidity absorption [20], analgesic [21], antitumor [22], and hemostatic [23]. Recent research demonstrated that chitosan nanoparticles have superior permeability, and immune modulation, with no toxicity to human cells [11], and enhanced antibacterial activity compared with bulk chitosan [24]. Furthermore, much research effort has been committed to the chitosan nanoparticles/metals hybrids development, to connect the chelating properties of chitosan with the antibacterial properties of metals. The chelation of metals by chitosan increases the positive charge density of chitosan nanoparticles leading to their enhanced adsorption onto the negatively charged bacterial cell wall, and therefore the inhibition of bacterial cell growth [25]. Zinc is one of these metals, showing great antibacterial properties [26]. Based on the clinical trials data, zinc also has antiseptic properties without harmful effects or a prolongation of the wound-healing process, especially in the case of burns, chronic wounds, and other infections [27]. An additional advantage of using chitosan and zinc for obtaining medical textiles is their approval by US-FDA as safe for many dietetic and wound dressing applications [28,29,30].

As bacterial infections rapidly circle the Earth, medical costs for their prevention and therapy skyrocket, with the medical waste stream continuously growing. From an environmental standpoint, it is preferable to manufacture durable, reusable, or biodegradable products. Taking into account antibacterial textiles, products preserving the antibacterial properties after washing would be of benefit, especially for high-value-added products for people with decubitus or sensitive skin susceptible to wounds, and a huge number of healthcare and hygiene products for use in hospitals [31]. With the aim of production of antibacterial textiles, many studies related to chitosan nanoparticles functionalization of different textiles (cotton [32], cotton/polyester blend [33], polyester [34], and silk [35,36]) have been presented; however, less attention has been paid to investigations related to washing durability of the obtained antibacterial textiles [37].

In order to obtain washing durable antibacterial activity by viscose fabric, irreversible immobilization of chitosan onto/into viscose is of the utmost importance. Similarities in the structure of viscose/cellulose and chitosan enable high affinity between both polymers, with intermolecular interactions mostly based on H-bonds and van der Waals forces [37]. However, enabling electrostatic attraction as well as covalent binding is required for more intense and irreversible binding between viscose and chitosan [1]. In this regard, it is necessary to introduce carboxyl (COOH) groups in the cellulose molecules since they provide electrostatic attraction, and/or aldehyde (CHO) groups as the most appropriate for covalent binding of chitosan by forming a Schiff base [38].

The introduction of COOH and CHO groups onto/into viscose fabric for more intense and irreversible binding of chitosan molecules can be achieved by two different pretreatments, i.e., by oxidation with 2,2,6,6-tetramethylpiperidine-1-oxy radical (TEMPO) carried under neutral condition, and by coating with TEMPO-oxidized cellulose nanofibrils (TOCN) obtained by ultrasonic defibrillation of TEMPO-oxidized cotton fibers, as was demonstrated in our previous study [32]. Thus, the viscose fabrics were pretreated, i.e., TEMPO-oxidized and coated with TOCN before functionalization with chitosan nanoparticles without and with embedded zinc ions (NCS and NCS + Zn, respectively) to enhance antibacterial activity, and even more desirable, preserve it after washing. According to a detailed literature review and the best of our knowledge, such functionalization of viscose fabrics (TEMPO-oxidation and coating with TOCN in combination with NCS and NCS + Zn deposition) and characterization of the products have not previously been reported. Chemical, electrokinetic, and antibacterial properties, as well as surface morphology of pristine and pretreated viscose fabrics functionalized with chitosan nanoparticles, were studied and compared. The viscose fabrics were thus characterized using Fourier transform infrared (FTIR) and X-ray photoelectron spectroscopy (XPS), elemental analysis, inductively coupled plasma optical emission spectrometry (ICP-OES), zeta potential measurements, and scanning electron microscopy (SEM). The COOH and CHO group content was determined by titration method, antibacterial activity was evaluated under dynamic contact conditions, and washing durability of the chitosan nanoparticles functionalized viscose fabrics was evaluated through changes in the nitrogen (i.e., chitosan) content and zinc content, electrokinetic properties, and antibacterial activity after one, three, and five washing cycles.

## 2. Materials and Methods

### 2.1. Materials

Regenerated cellulose fabric “15A23 viscose uni Sandy–white” with a surface mass of the fabric: 82 g/m^2^, yarn count: 9.6 tex × 9.9 tex, fabric count: 400 warp threads/10 cm and 350 weft threads/10 cm, purchased from IGR Agence, Celje, Slovenia, was used as a starting material. Chitosan (CS) with low molecular weight (Aldrich, 448869), 75–85% deacetylated, hydrochloric acid (HCl), TEMPO, sodium bromide (NaBr), zinc acetate dehydrate (ZnAc•2H_2_O), sodium tripolyphosphate (TPP), sodium chlorite (NaClO_2_), sodium hydroxide (NaOH), 13% sodium hypochlorite (NaClO), 30% hydrogen peroxide (H_2_O_2_), and 65% nitric acid (HNO_3_) were from Sigma-Aldrich (Vienna, Austria). All chemicals were of analytical grade and used without further purification.

### 2.2. Preparation of NCS Dispersion

A CS solution (0.5% *w*/*v*) was prepared by suspending CS in distilled water; thereafter, suspension pH was adjusted to 2.5 by addition of 1 M HCl. The resulting suspension was stirred using a laboratory magnetic stirrer for 24 h at room temperature until complete dissolution of the CS. TPP solution (1 mg/mL) was prepared by dissolving TPP in distilled water. Finally, NCSs were formed spontaneously by dropping TPP solution into the CS solution (in a ratio of 1:1 (*w*/*w*)) during 1 h solution stirring at room temperature. The NCS dispersion pH was adjusted to pH 5.5 by addition of 0.5 M NaOH.

### 2.3. Preparation of NCS + Zn Dispersion

NCS + Zn dispersion was prepared by dropping 3% solution of ZnAc into NCS dispersion prepared according to the above-described method, in a ratio of 10:1 (*w*/*w*). The NCS + Zn dispersion pH was adjusted to pH 5.5 by addition of 0.5 M NaOH.

### 2.4. Preparation of TEMPO-Oxidized Cellulose Nanofibrils

TOCN was prepared by defibrillation of TEMPO-oxidized cotton fibers according to the method reported by Korica et al. [37].

### 2.5. Pre-Treatment of Viscose Fabric

In order to introduce COOH and CHO groups onto/into viscose fabric to achieve more intense and irreversible binding of NCS and NCS + Zn, viscose fabric was pretreated by:

(a) TEMPO-oxidation performed under neutral condition, at pH 7, according to the procedure described by Korica et al. [37], and
(b) Coating with 0.5% (*w*/*v*) TOCN dispersion according to the procedure described by Korica et al. [37].

Pretreated samples were conditioned at 20 ± 2 °C, and relative humidity of 65 ± 4%, before further processing or characterization.

### 2.6. Functionalization of Viscose Fabrics with NCS and NCS + Zn

The pristine and pretreated viscose fabrics were immersed into the NCS or NCS + Zn dispersion for 30 min, at room temperature, with a material–liquid bath ratio of 1:50. The excess liquid was removed from the samples by squeezing at a pressure of 2 bars onto a laboratory padder (Rapid, Istanbul, Turkey) to a wet pick up of 100%; functionalized fabrics were dried at 40 °C for 30 min in a laboratory oven (Instrumentaria, Zagreb, Croatia) and conditioned at 20 ± 2 °C with relative humidity of 65 ± 4% before characterization.

### 2.7. Washing of Functionalized Viscose Fabrics

Functionalized viscose fabrics were washed according to standard ISO 105-C10. The sample denotations of viscose fabrics before and after washing are listed in Table 1.

### 2.8. Determination of COOH and CHO Group Content

COOH and CHO group content in pristine and pretreated viscose fabrics were determined by the modified calcium acetate method described by Praskalo et al. [40].

### 2.9. FTIR Analysis

FTIR spectra in the wavenumber range of 600–4000 cm^−1^, at a resolution of 2 cm^−1^, and in 20 scan mode, were recorded using Shimadzu IRA Infinity-1 (FT-IR) spectrophotometer (Shimadzu Corporation, Kyoto, Japan) equipped with attenuated total reflectance accessory (ATR) using a diamond/ZnSe crystal. Prior to ATR-FTIR measurements, samples were dried at 40 °C for 24 h and stored in the desiccator until the analysis.

### 2.10. X-ray Photoelectron Spectroscopy (XPS)

The surface chemical analysis of the samples was performed using a PHI TFA XPS from Physical Electronics, Chanhassen, MN, USA. The base pressure in the XPS analysis chamber was approximately 6 × 10^−8^ Pa. The samples were excited with X-rays over a 400 µm area with monochromatic AlKα_1,2_ radiation at 1486.6 eV, operating at 200 W. Photoelectrons were detected with a hemispherical analyzer (Physical Electronics, Chanhassen, MN, USA), positioned at an angle of 45° to the sample surface. A detection depth was approximately a few nm. Spectra were measured at least at two different locations on each sample. Survey spectra were taken at a pass-energy of 187 eV and a 0.4 eV energy step. High-resolution spectra were acquired at a pass-energy of 23.5 eV and a 0.1 eV energy step. An additional electron gun was used to compensate the surface charge accumulation during the measurement. Binding energy was calibrated by setting the C1s peak at 284.8 eV. The MultiPak software (Physical Electronics, Chanhassen, MN, USA) was used to calculate the surface elemental concentrations from the survey scan spectra.

### 2.11. Elemental Analysis

The nitrogen percentage in the NCS and NCS + Zn functionalized viscose fabrics was determined by the elemental analysis performed on a Vario EL III C,H,N,S/O Elemental Analyzer (Elementar Analysensysteme GmbH, Langenselbold, Germany). Subsequently, the chitosan content in the NCS and NCS + Zn functionalized viscose fabrics was calculated based on the nitrogen percentage.

### 2.12. ICP-OES Analysis

ICP-OES and microwave sample digestion were utilized to determine the zinc content in the NCS + Zn functionalized viscose fabrics. The digestion was performed on an advanced microwave digestion system (ETHOS 1, Milestone, Italy) using an HPR-1000/10S high-pressure segmented rotor. The pressure-resistant PTFE vessels (volume 100 mL) with a fluoropolymer liner were used in this study. Prior to use, the PTFE vessels were cleaned with acid and rinsed with deionized water. In each of the clean vessels, about 0.25 g of precisely weighed sample was mixed with a mixture of 8 mL HNO_3_ (65%) and 1 mL H_2_O_2_ (30%), and heated with microwave energy for 20 min at 180 °C.

ICP-OES analysis was performed using a Thermo Scientific iCAP 6500 Duo ICP (Thermo Fisher Scientific, Cambridge, UK) spectrometer equipped with RACID86 Charge Injector Device (CID) detector, standard glass concentric nebulizer, quartz torch, and alumina injector. The optical system was purged with argon and the Echelle polychromator was thermostated at 38 °C. Multi-elemental plasma standard solution (Multi-Element Plasma Standard Solution 4, Specpure^®^, 1000 µg·mL^−1^) certified by Alfa Aesar GmbH & Co KG, Karlsruhe, Germany, was used to preparing calibration solutions for ICP-OES analysis. The analyses were performed on Zn I 213.856 nm emission lines. Two types of blanks were required for the analysis of the prepared samples. The calibration blank was used to establish the analytical curve, while the method blank was used to identify possible contamination originating from either the reagents (acids) or the equipment used during sample processing. For all digested samples, the ICP-OES analysis was performed in triplicate.

### 2.13. The Zeta Potential Measurement

The zeta potential of viscose fabrics was determined using a SurPASS electrokinetic analyzer (Anton Paar GmbH, Graz, Austria) according to the streaming potential method described by Korica et al. [37].

### 2.14. SEM Analysis

JEOL JSM-5300 scanning electron microscope (JEOL, Tokyo, Japan) was used to investigate the surface morphology of viscose fibers Before SEM analysis. The samples were sputtered with gold using IONSPUTTER, JEOL, model JFC-1100E (JEOL, Tokyo, Japan).

### 2.15. Antibacterial Activity Determination

The antibacterial activity of viscose fabrics was determined against Gram-negative bacteria *Escherichia coli* ATCC 25922 (*E. coli*) and Gram-positive bacteria *Staphylococcus aureus* ATCC 25923 (*S. aureus*) using a standard method for testing the antimicrobial activity of immobilized antimicrobial agents under dynamic contact conditions ASTM E 2149-01 (2001). The bacterial reduction (*R*, %) was calculated by the following equation:

(1) $$R=\frac{C_{0}-C}{C_{0}}\times 100\%$$

where *C*_0_ (CFU) presents the number of bacteria colonies on the control sample and *C* (CFU) presents the number of bacteria colonies on the functionalized viscose fabric.

## 3. Results and Discussion

### 3.1. Physicochemical and Antibacterial Properties of Viscose Fabrics

**Figure 1:**
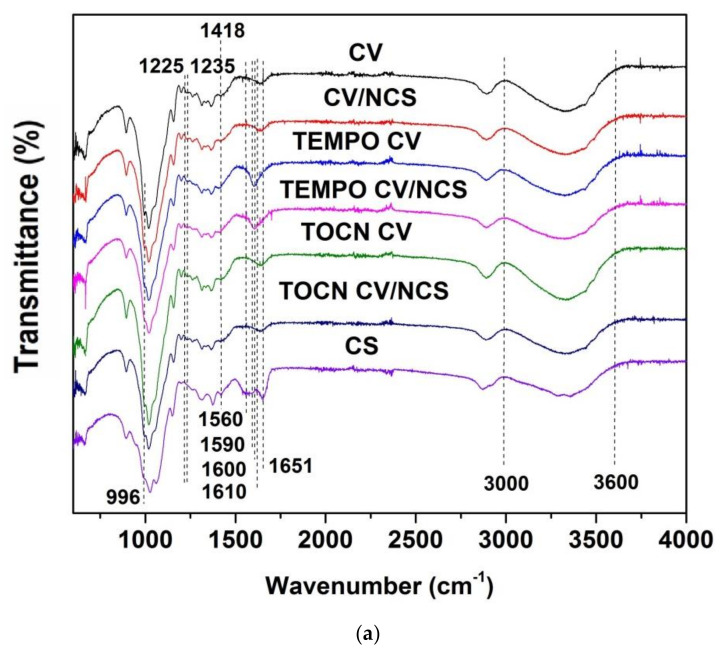

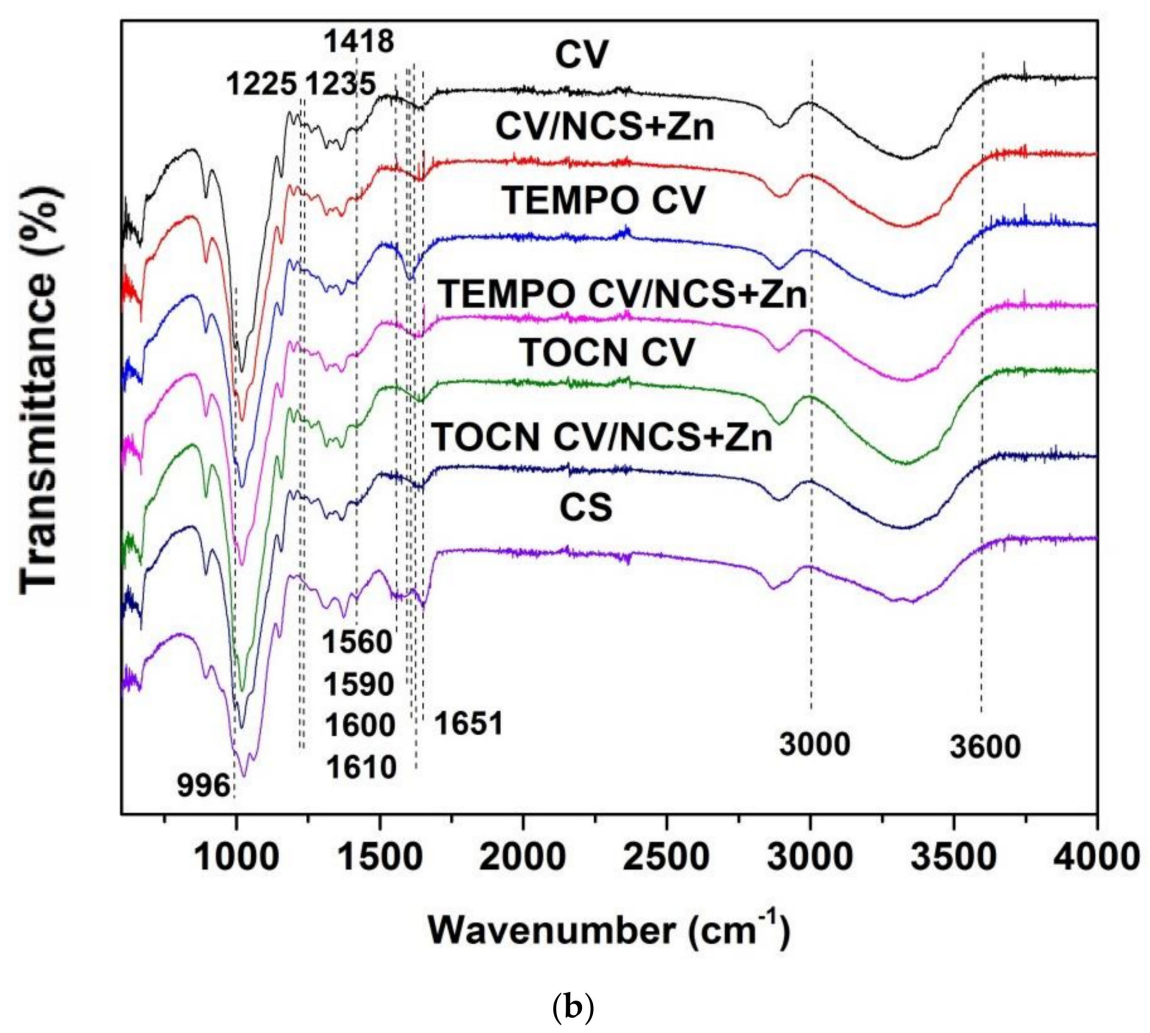
FTIR spectra of (**a**) NCS and (**b**) NCS + Zn functionalized viscose fabrics, modified from [39].

The FTIR-ATR spectra recorded to characterize the surface of pristine, pretreated, NCS and NCS + Zn functionalized viscose fabrics include characteristic bands of cellulose (Figure 1): at 892–896 cm^−1^ is the band corresponding to C–O–C valence vibration of β-glycosidic linkage or C1-H deformation in cellulose II; at 996 cm^−1^ is the band related to C–O valence vibration at C6; the band at 1024 cm^−1^ is characteristic for C–O stretching; the band at 1067 cm^−1^ corresponds to C–O vibration mainly from C3–O3H in cellulose II; the band at 1158 cm^−1^ is related to C–O–C asymmetric valence vibration from β-glycosidic linkage in cellulose II; the bands at 1200 and 1336 cm^−1^ originate from OH in-plane deformation; the band at 1225–1235 cm^−1^ is due to O–H in-plain deformation at C6; the band at 1316 cm^−1^ is related to CH_2_ wagging vibration; the band at 1372 cm^−1^ is due to C–H deformation in cellulose II; the band at 1418 cm^−1^ corresponds to CH_2_ scissoring at C6 in cellulose II; the broad band at 1636 cm^−1^ is due to the adsorbed water; the band at 2893 cm^−1^ originates from C–H stretch in cellulose II and deformation vibrations of CH_2_, CH_2_OH in cellulose from C6; and the wide band at 3000–3600 cm^−1^ is characteristic for hydrogen-bonded OH groups [41,42].

TEMPO-oxidation and coating with TOCN were used to introduce new functionalities, i.e., carboxyl and aldehyde functional groups, into/onto pristine viscose fabrics. As we reported earlier [37], TEMPO-oxidation leads to oxidation of the primary hydroxyl groups of glucopyranose units into carboxylate groups via aldehyde intermediates, and thus to the increment of carboxyl (0.438 mmolg^−1^) and aldehyde (0.440 mmolg^−1^) functional group content in TEMPO-oxidized viscose compared with pristine viscose fabric (0.064 and 0.018 mmolg^−1^, respectively). In the case of coating with TOCN, the increment of carboxyl (0.086 mmolg^−1^) and aldehyde (0.027 mmolg^−1^) functional group content is less pronounced. The appearance of a new band at 1600 cm^−1^ in the FTIR-ATR spectrum of TEMPO CV, corresponding to C=O stretching vibration, confirms conversion of hydroxyl groups at the C6 position of cellulose molecules into sodium carboxylate groups by TEMPO-oxidation [43,44]. The same band in the spectrum of TOCN CV did not appear probably due to the low content of the sodium carboxylate groups, and the fact that this band can be masked by the broadband of adsorbed water, which is located in the same region of the spectrum. Concerning the aldehyde groups, there is no classical adsorption of these groups in the FTIR spectra since they are partially or even completely hydrated forming hemiacetal or hemialdol structures. Compared with pristine viscose, FTIR spectra of TEMPO-oxidized and TOCN-coated viscose show only a slight decrease in the intensity of the bands at 996 cm^−1^ (C–O valence vibration at C6), 1225–1235 cm^−1^ (O–H in-plain deformation at C6), and 1418 cm^−1^ (CH_2_ scissoring at C6 in cellulose II).

The FTIR spectra of NCS (Figure 1a) and NCS + Zn (Figure 1b) functionalized viscose fabrics show characteristic bands for cellulose and chitosan with slight modifications. By comparing their spectra with the spectrum of pure chitosan, it is evident that the major bands of chitosan (i.e., bands related to NH_2_ stretching vibration at 1560 cm^−1^, N-H bending of the amide II at 1590 cm^−1^, and carbonyl stretching of acetyl groups at 1651 cm^−1^ [45,46]) almost disappeared after NCS and NCS + Zn functionalization of viscose fabrics. The reason for this could be hydrogen bonding between viscose and chitosan nanoparticles, but also the fact that the broadband originating from adsorbed water masks their signal. The former was confirmed by the decreased intensity and a slight shift to higher wavenumbers of the broadband between 3000–3600 cm^−1^ related to the O-H stretching vibrations overlapped with NH_2_ stretching [47]. Additionally, in the case of viscose fabric highly decorated with negatively charged carboxylate groups (TEMPO CV sample), the electrostatic interactions (ionic binding) between these groups of TEMPO CV and positively charged ammonium groups of NCS and NCS + Zn, as well as zinc ions, was confirmed by a slight shift of the band assigned to C=O stretching vibration of the carboxylate groups to higher wavenumbers (from 1600 to 1610 cm^−1^) and by a significant decrease in its intensity [48,49,50]. However, the reaction of aldehyde groups present in viscose fabrics with amino groups present in NCS and NSC + Zn and formation of a Schiff base cannot be confirmed since the characteristic band corresponding to these covalent bonds could be masked by the inevitable absorbance of water bound to cellulose and chitosan.

**Figure 2:**
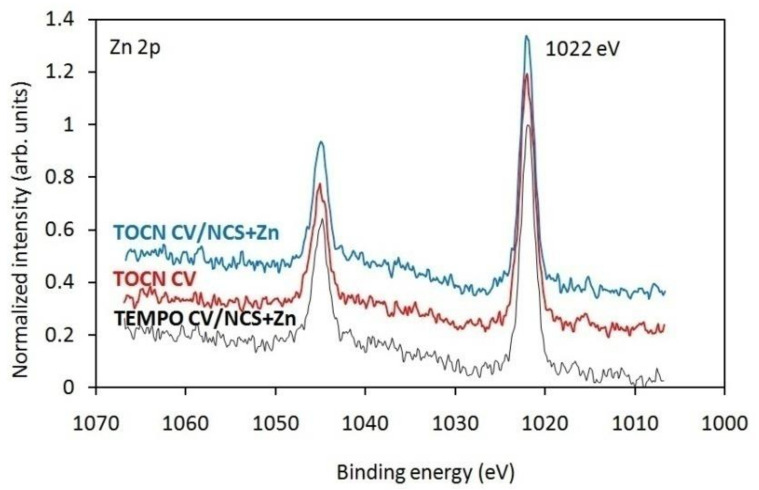
High-resolution spectra of NCS + Zn functionalized pristine and pre-treated viscose fabrics.

XPS analysis, as a selective and sensitive technique for surface characterization, was performed to determine the surface composition of NCS + Zn functionalized viscose fabrics and to clarify the form of zinc in NCS + Zn functionalized viscose fabrics. Survey spectra, which are shown in Figure S1 in the Supplementary Material, were measured to determine the elemental composition. For all samples, the survey spectra show the presence of peaks corresponding to carbon, oxygen, and nitrogen. However, for NCS + Zn functionalized pristine and pre-treated viscose fabrics the XPS survey spectra also clearly show the presence of zinc thus confirming the successful attachment of zinc-containing nanoparticles to the surface. High-resolution spectra of Zn 2p are additionally presented in Figure 2. Doublet peaks of Zn 2p_3/2_ and Zn 2p_1/2_ are positioned at binding energies of approximately 1022 eV and 1045 eV, respectively, indicating that the Zn in the NCS + Zn functionalized viscose fabrics is in the form of Zn-O species [51]. However, according to Perelshtein et al. [52], no individual CS–ZnO and CS–Zn^2+^ complexes can be reliably distinguished since zinc shows only a small shift of binding energy in the Zn 2p_3/2_ region.

Elemental surface compositions of the pristine and pre-treated viscose fabrics before and after functionalization with NCS + Zn determined by XPS are presented in Table 2. The highest atomic percentage of N and Zn was obtained for NCS + Zn functionalized TEMPO-oxidized viscose fabric (sample TEMPO CV/NCS + Zn), while similar values were obtained for NCS + Zn functionalized pristine (sample CV/NCS + Zn) and TOCN-coated viscose (sample TOCN CV/NCS + Zn). Elements present at the trace level are impurities or residual chemicals from different functionalization steps.

Since the depth of XPS analysis is below 10 nm, i.e., obtained results are related to only a certain number of molecular layers on the fabric surface, the elemental analysis was used to quantify chitosan and zinc content in the bulk of viscose fabrics after functionalization with NCS/NCS + Zn (Table 3). Compared with pristine viscose fabric, both pretreated viscose fabrics have a higher chitosan content (i.e., 2.74, 1.47, 1.65, and 1.59 times higher for TEMPO CV/NCS, TOCN CV/NCS, TEMPO CV/NCS + Zn, and TOCN CV/NCS + Zn, respectively), which can be attributed to higher functional group content in these fabrics. Furthermore, NCS + Zn functionalized pretreated viscose fabrics have increased zinc content, i.e., 1.83 and 1.10 times higher for TEMPO CV/NCS + Zn and TOCN CV/NCS + Zn, respectively, compared with NCS + Zn functionalized pristine CV. The highest CS and Zn content achieved for TEMPO-oxidized viscose fabrics is directly related to the highest functional group content, i.e., 0.878 mmol of COOH + CHO per g of TEMPO CV vs. 0.113 mmol of COOH + CHO per g of TOCN CV or 0.082 mmol of COOH + CHO per g of CV. Based on the fact that the ratio between zinc and chitosan contents is different for all NCS + Zn functionalized fabrics, it can be concluded that total zinc content in fabrics does not originate only from NCS + Zn, i.e., a certain amount of dissociated zinc ions from initial NCS + Zn dispersion (as a coating) was bounded into/onto fabrics during functionalization procedure. The latter is the most pronounced for sample TEMPO CV/NCS + Zn, which might be explained by its very high carboxyl groups’ content and increased electrostatic attraction between them and zinc ions present in liquid medium resulting in the highest amount of zinc directly bounded into/onto fabric in the case of TEMPO CV/NCS + Zn. Finally, in the case of NCS + Zn functionalized pristine and pretreated viscose fabrics, the zinc concentration determined by the XPS is lower than the concentration determined by the ICP-OES, while the nitrogen concentration determined by the XPS is higher than the concentration determined by the ICP-OES. These indicate that chitosan nanoparticles are located on the viscose fabric surface, while a certain amount of zinc is incorporated within the viscose fibers below the 10 nm surface depth scanned by the XPS.

**Figure 3:**
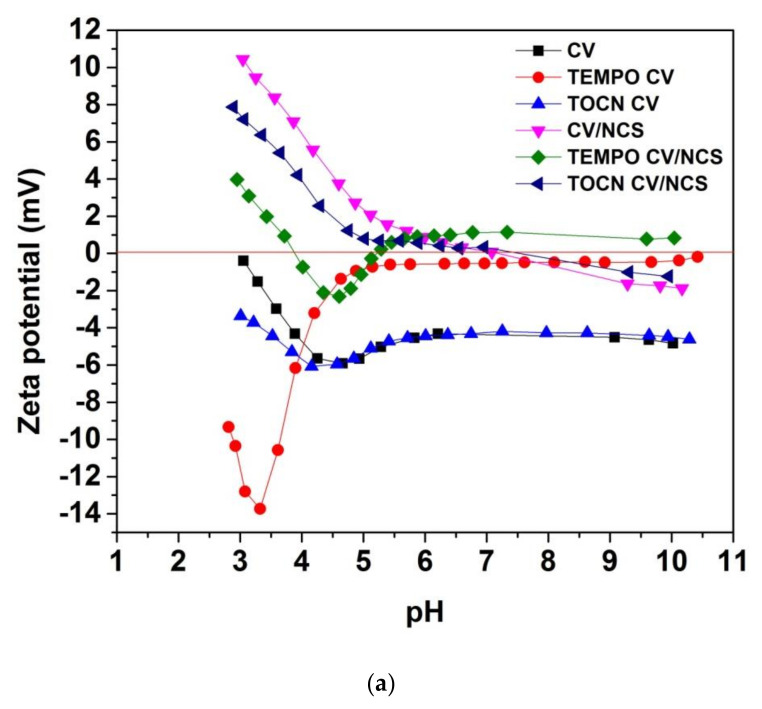

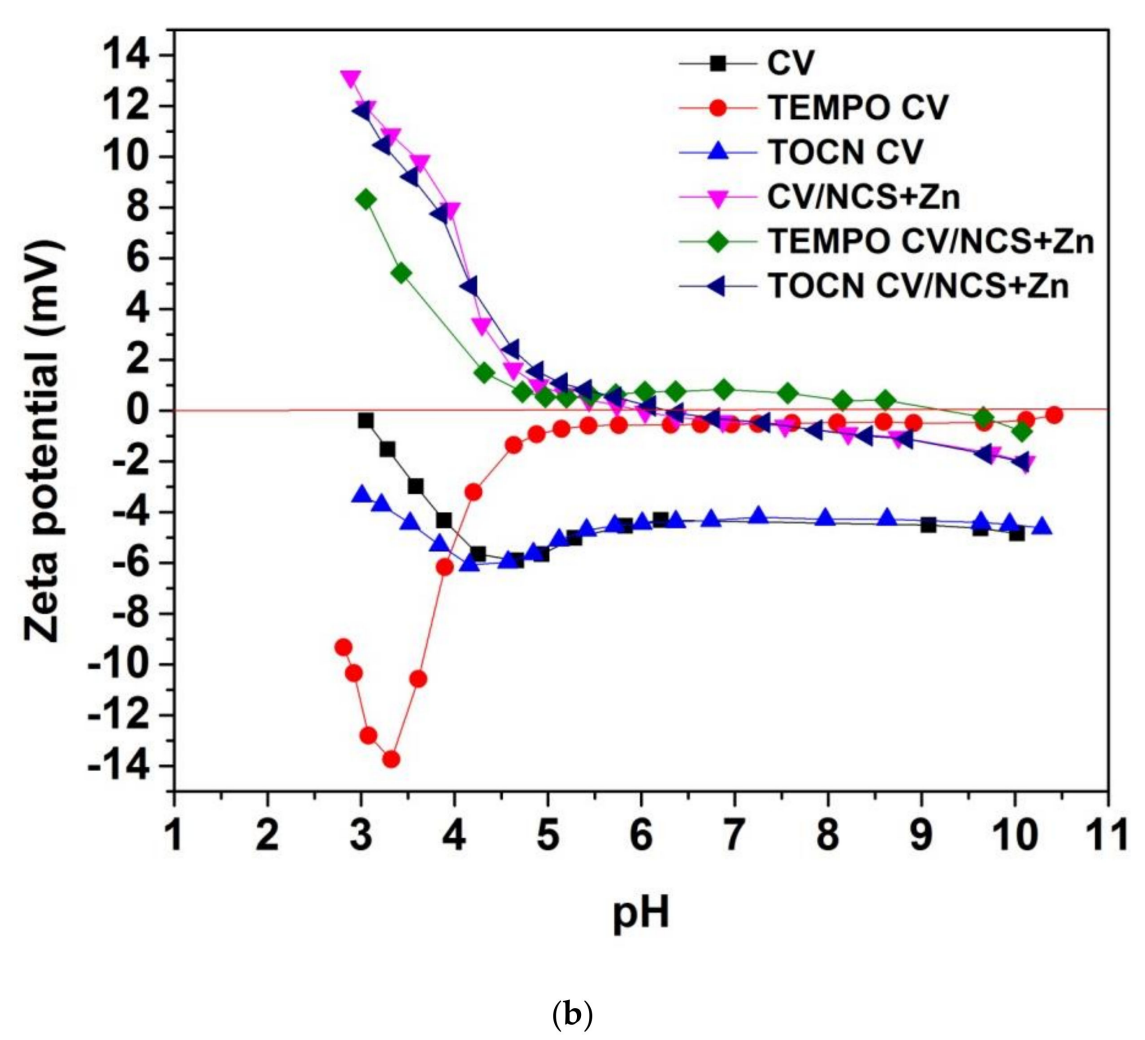
Zeta potential of (**a**) NCS and (**b**) NCS + Zn functionalized viscose fabrics, modified from [39].

Zeta potential (ζ) measurements give information about the surface charge as a function of pH and indirectly about surface acidic-base functional groups and their reactivity in an aqueous medium allowing comparison with the surface charge behavior of materials prior to and after their surface treatments [53]. Figure 3 shows pH-dependent zeta potential curves of viscose fabrics before and after NCS and NCS + Zn functionalization. Pristine and pretreated viscose fabrics (samples CV, TEMPO CV, and TOCN CV) have negative zeta potential overall in the investigated pH region (Figure 3) owing to the presence of anionic functionalities on their surface (i.e., carboxyl groups’ ionization and hydroxyl groups’ specific adsorption), as was demonstrated in our previous study [37]. As a consequence of augmented acidic group content on the viscose fabric surface after pretreatments, the isoelectric point (IEP) was shifted from 2.94 for CV to 1.74 for TEMPO CV and to 1.81 for TOCN CV. However, for CV and TOCN CV samples, the quotient of the maximum negative zeta potential (ζ_max_) and the plateau value (ζ_plateau_), related to materials swelling, is similar, confirming that coating with TOCN did not affect the inner structure of viscose. On the other hand, increased ζ_max_/ζ_plateau_ and decreased ζ_plateau_ for TEMPO-oxidized viscose indicate its enhanced hydrophilicity and swelling due to the changed inner structure of viscose fibers provoked by TEMPO-oxidation. Furthermore, such changes lead to enhanced accessibility of the inner fiber structure and an enlarged total fiber surface, especially in the swollen state, increasing the capacity of TEMPO CV to bind chitosan nanoparticles, and zinc ions (Table 3).

After functionalization of viscose fabrics with NCS and NCS + Zn, their IEPs are shifted to higher pH values, while the whole zeta potential-pH curves are moved to higher values (Figure 3). At the alkaline pH range, higher values of zeta potential for functionalized fabrics indicate a lower amount of accessible acidic groups because of the deposition of NCS or NCS + Zn on the fiber, i.e., fabric surface. Since the protonation of chitosan’s amino groups at alkaline pH range is suppressed (IEP of chitosan is about 6.8 [54]), the obtained negative zeta potential values for NCS and NCS + Zn functionalized pristine and TOCN-coated viscose fabrics were expected. However, slightly positive values of zeta potential at alkaline pH range obtained for NCS and NCS + Zn functionalized TEMPO-oxidized viscose fabrics can be explained by charge reversal [37,55] attributed to strongly adsorbed cations such as sodium ions, which are counterions of carboxylate groups in TEMPO-oxidized viscose, and zinc ions present in NCS + Zn dispersion used for functionalization. At acidic pH conditions, the surface of functionalized fabrics is positively charged because of suppressed dissociation of cellulose acidic groups and enhanced protonation of chitosan amino groups. In the whole pH range, NCS + Zn functionalized fabrics show higher zeta potential values compared with NCS functionalized fabrics due to the presence of zinc ions onto fabrics’ surfaces and the fact that zinc ions increase the positive charge density of NCS; the shift of IEP from 8.02 for NCS to 8.86 for NCS + Zn (Figure S2, in the Supplementary Material) confirms that chelation of zinc with NCS increases the positive charge density of NCS with embedded zinc ions.

**Figure 4:**
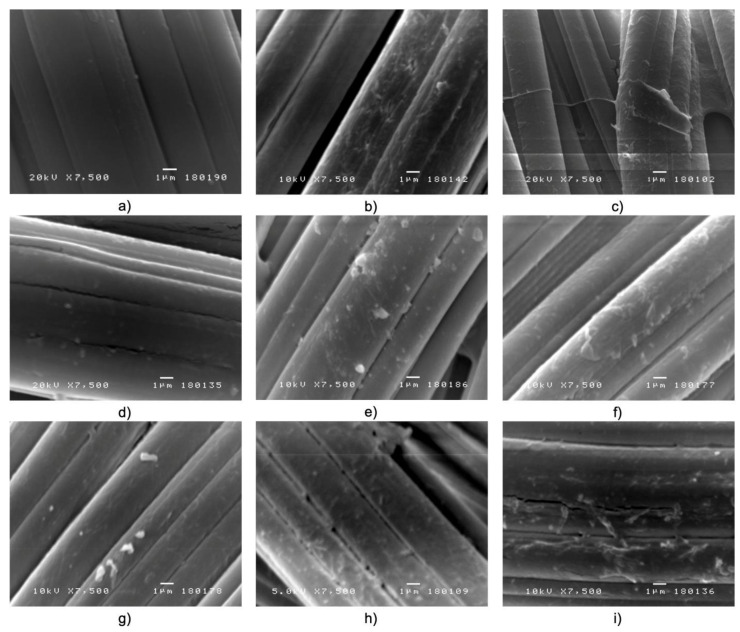
SEM images of CV (**a**), TEMPO CV (**b**), TOCN CV (**c**), CV/NCS (**d**), TEMPO CV/NCS (**e**), TOCN CV/NCS (**f**), CV/NCS + Zn (**g**), TEMPO CV/NCS + Zn (**h**), TOCN CV/NCS + Zn (**i**).

The surface morphology of the viscose fibers/fabrics before and after functionalization with NCS and NCS + Zn is shown in Figure 4. Besides the introduction of CHO and COOH groups into cellulose, TEMPO-oxidation brings about structure damages in the form of cracks on the fiber surface (Figure 4b) making it more open and consequently more efficient for immobilization of chitosan nanoparticles and zinc ions (in the case of NCS + Zn functionalized fabrics). From Figure 4c, it can be seen that viscose fibers are coated with the TOCN thin film. The influence of the surface morphology and chemistry of pristine and pretreated viscose fabrics on the adsorption degree of NCS and NCS + Zn can be evaluated by analyzing together data for chitosan content (Table 3) and the surface morphology of the NCS and NCS + Zn-coated fibers (Figure 4d–i). This analysis confirms that pretreated viscose fiber surfaces dictate more efficient adsorption, whereby TEMPO-oxidized viscose fibers represent the most efficient immobilization surface for an enhanced attachment of NCS and NCS + Zn. From Figure 4d–i, it can clearly be seen that pretreated viscose fibers are more densely covered by NCS and NCS + Zn than their pristine counterparts. The densest coverage by NCS and NCS + Zn, which is also populated with their largest agglomerates, was noticed for TEMPO-oxidized viscose fibers.

The bacterial reduction of NCS and NCS + Zn functionalized viscose fabrics were determined against Gram-positive (*S. aureus*) and Gram-negative (*E. coli*) bacteria. According to the used standard, the fabrics show antibacterial activity in the case of bacterial reduction higher than 75% [56]. The maximum bacterial reduction of both tested bacteria was obtained for all functionalized fabrics (Table 4). An exception to this was observed for CV/NCS, i.e., fabric with the lowest chitosan content, for which sufficient but a slightly lower bacterial reduction of *E. coli* was observed. Slightly better bacterial reduction of *S. aureus* compared with *E. coli* for CV/NCS can be explained by a more complex bacterial cell surface structure, i.e., an outer membrane presence in *E. coli* acting as a barrier to environmental influence, that is already reported [57].

Recent studies propose four main mechanisms of chitosan action against microbes: (i) disrupting the cell membrane/wall, (ii) interaction with microbial DNA, (iii) nutrients’ chelation by chitosan, and (iv) formation of an external polymer barrier on the cell surface [58]. It is accepted that all mechanisms occurred simultaneously, whereby mechanisms based on disrupting the cell membrane/cell wall have a dominant effect [59]. On the other hand, Zn^2+^ ions’ antimicrobial activities were explained by two mechanisms: (i) disrupting the cell membrane/cell wall, and (ii) interaction with microbial DNA [60]. Given that chitosan chelates metals [61], the synergetic antimicrobial activity of chitosan and Zn^2+^ ions unquestionably depends on the release of Zn^2+^ ions from NCS + Zn [62]. In NCS + Zn functionalized viscose fabrics a certain amount of Zn^2+^ ions is directly bounded into/onto the viscose fabrics, so the synergetic antimicrobial activity of chitosan nanoparticles with embedded Zn^2+^ ions is additionally ameliorated by releasing these Zn^2+^ ions. Furthermore, the antibacterial activity of chitosan nanoparticles chelated with metal ions directly proportional to zeta potential [63] was confirmed. From Figure S2 it can be seen that chelation of chitosan nanoparticles with Zn^2+^ ions increases the positive charge density of NCS + Zn, while from Figure 4 it can be seen that viscose fabrics functionalized with NCS + Zn have increased positive charge density compared with viscose fabrics functionalized with NCS. These increases in positive charge density were expected to lead to intensification of the mechanism based on disrupting the cell membrane/cell wall which is common for both antimicrobial compounds used, i.e., chitosan and Zn^2+^ ions. Comparing bacterial reduction of *E. coli* for CV/NCS and CV/NCS + Zn samples, it is obvious that an increase in positive charge density due to the addition of zinc ions for all NCS + Zn functionalized fabrics (Figure 4) leads to more intense electrostatic interactions with negatively charged components of the bacterial cell wall and inhibition of bacterial cell growth. The attained antibacterial activity against both tested strains was similar to those reported for chitosan functionalized, in the same manner as pretreated viscose fabrics samples [37], but much higher compared with viscose functionalized in a similar way [1,13], for which only satisfying antibacterial activity against *S. aureus* was achieved.

### 3.2. Washing Durability of Chitosan Nanoparticles Functionalized Viscose Fabrics

Chitosan nanoparticles functionalized viscose fabrics washing durability towards multiple washing (i.e., one, three, and five washing cycles) was assessed through changes in their electrokinetic surface properties (zeta potential), chitosan and zinc content, and antibacterial activity.

**Figure 5:**
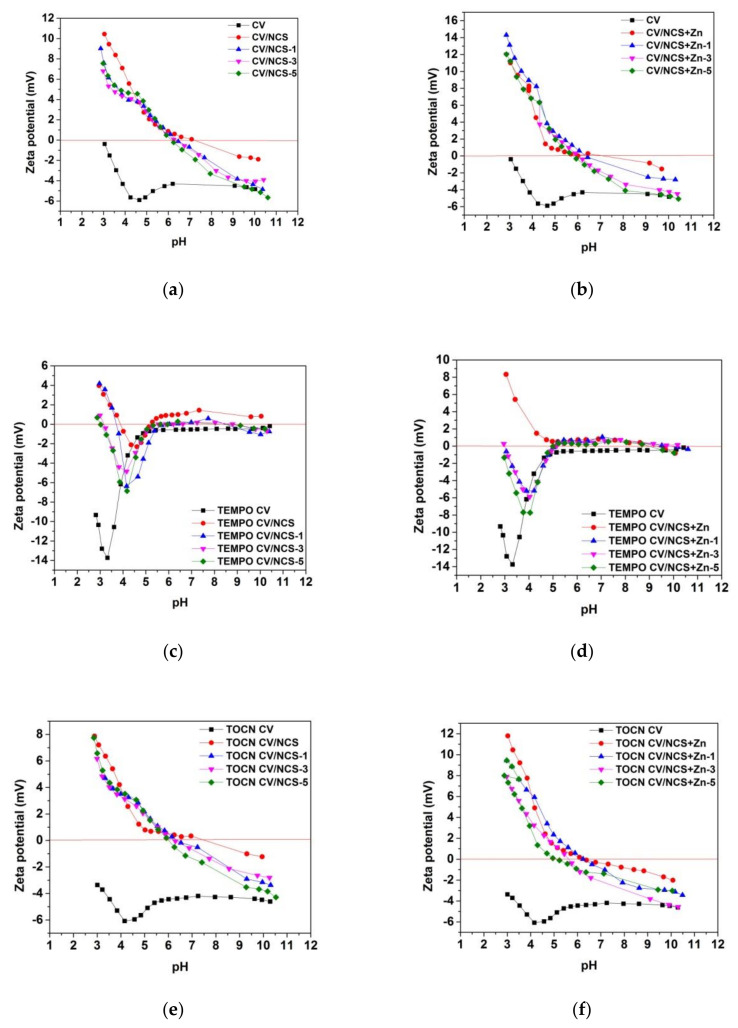
Zeta potential of NCS (**a**,**c**,**e**) and NCS + Zn (**b**,**d**,**f**) functionalized viscose fabrics, before and after 1, 3 and 5 washing cycles; modified from [39].

The changes in the zeta potential of chitosan nanoparticles functionalized viscose fabrics after multiple washing cycles (Figure 5) indicate the release of reversibly and/or weakly bound chitosan nanoparticles and also zinc ions, in the case of NCS + Zn functionalized fabrics, during the washing process; after each washing cycle IEPs were shifted to a lower pH. However, by comparison of the zeta potential-pH curves obtained for pristine and pretreated viscose fabrics and the NCS/NCS + Zn functionalized fabrics after five washing cycles, it is clear that NCS/NCS + Zn and zinc ions (for NCS + Zn functionalized fabrics) are still present on fabrics’ surfaces. Furthermore, from Figure S3, it can be seen that a shift in IEPs of all functionalized fabrics to lower pH is correlated with a decrease in chitosan and zinc ions content after each washing cycle.

**Figure 6:**
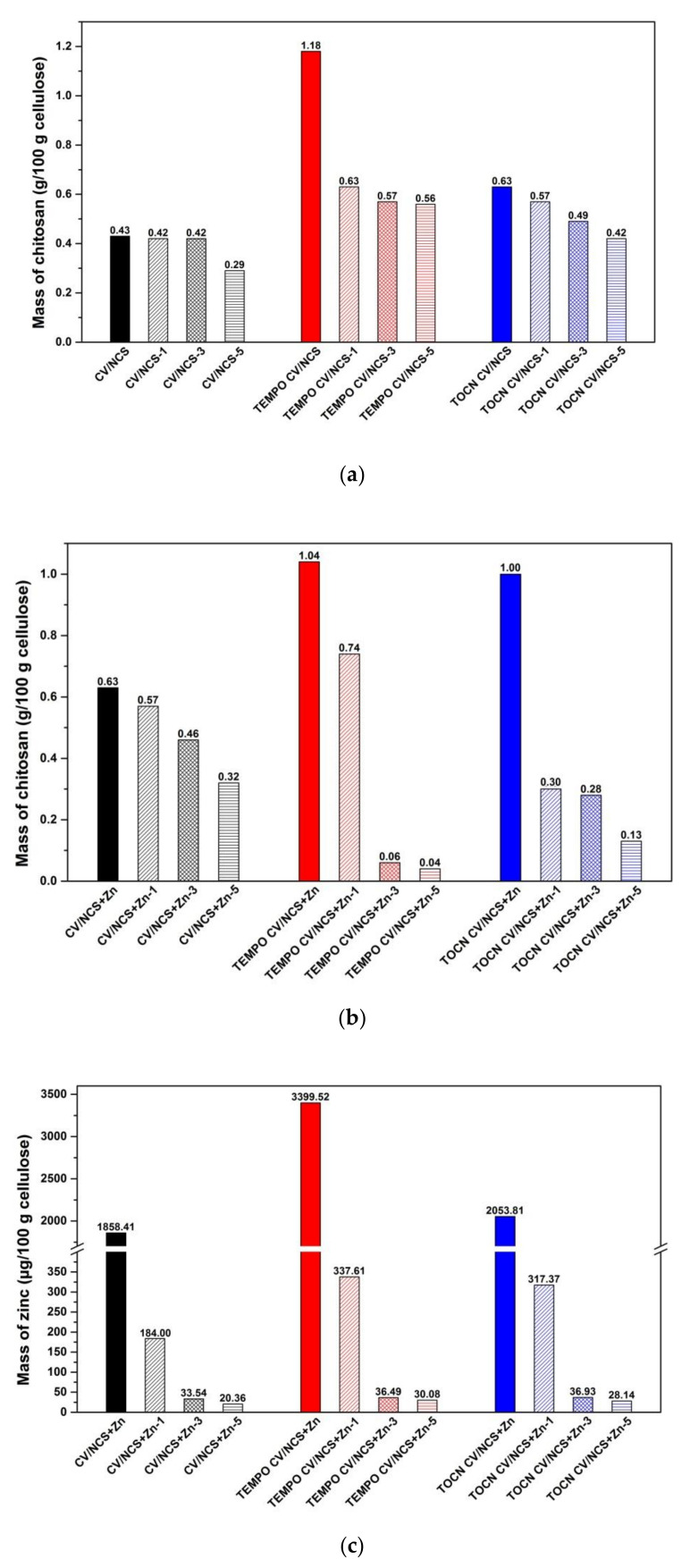
The chitosan (**a**,**b**) and zinc (**c**) content in the NCS and NCS + Zn functionalized viscose fabrics after 1, 3 and 5 washing cycles; modified from [39].

The chitosan and zinc content in the NCS and NCS + Zn functionalized viscose fabrics after one, three, and five washing cycles are presented in Figure 6. CV/NCS sample showed almost no changes in chitosan content after three washing cycles, while a total decrease in chitosan content of 33% after five washing cycles occurred. By TEMPO CV/NCS sample a significant decrease in chitosan content was determined already after first washing cycle (47%), followed by an additional decrease of 10% after the third washing cycle and only an additional 2% after the fifth washing cycle. A moderate and uniform decrease in chitosan content was noticed for TOCN CV/NCS sample, i.e., 10% after the first washing cycle, an additional 14% after the third washing cycle, and 14% after the fifth washing cycle. After five washing cycles, CV/NCS and TOCN CV/NCS samples showed similar washing durability with a total decrease in chitosan content of about 33%, while TEMPO CV/NCS sample was less durable to washing with a total decrease in chitosan content of about 53%. However, the remaining content of chitosan was the highest for TEMPO CV/NCS sample (0.56 g/100 g cellulose), lower for TOCN CV/NCS sample (0.42 g/100 g cellulose), and the lowest for CV/NCS (0.29 g/100 g cellulose) after five washing cycles. Compared with viscose fabrics functionalized with bulk chitosan, which were investigated in our previous study [37], viscose fabrics functionalized with NCS were characterized with better washing durability. Enhanced washing durability is especially observed in the cases of pristine and TOCN-coated viscose fabrics, namely CV/NCS sample has 45% lower total decrease in chitosan content than pristine viscose fabric functionalized with bulk chitosan, while TOCN CV/NCS sample has 24% lower total decrease in chitosan content than TOCN-coated viscose fabric functionalized with bulk chitosan.

Considering NCS + Zn functionalized viscose fabrics, the CV/NCS + Zn sample showed moderate decrease in chitosan content, i.e., 10% after the first washing cycle and 19% after the third washing cycle, and a higher additional decrease of 30% after the fifth washing cycle. The TEMPO CV/NCS + Zn sample showed a very high decrease in chitosan content after one and three washing cycles (29% after the first washing cycle and 92% after the third washing cycle), with a total decrease in chitosan content of 96% after five washing cycles. In the case of the TOCN CV/NCS + Zn sample, a significant decrease in chitosan content was determined after the first washing cycle (70%), a slight additional decrease after the third washing cycle (7%), and again, a significant additional decrease after the fifth washing cycle (54%). Taking into account the results after five washing cycles, the CV/NCS + Zn sample showed the highest washing durability (the total decrease in chitosan content of about 49%), TOCN CV/NCS + Zn sample showed lower (the total decrease in chitosan content of about 87%), while TEMPO CV/NCS + Zn sample showed the lowest washing durability (the total decrease in chitosan content of about 96%). The same trend was noticed for the remaining content of chitosan: CV/NCS + Zn sample (0.32 g/100 g cellulose) > TOCN CV/NCS + Zn sample (0.13 g/100 g cellulose) > TEMPO CV/NCS + Zn sample (0.04 g/100 g cellulose).

Comparing viscose fabrics functionalized with NCS, for viscose fabrics functionalized with NCS + Zn, a higher chitosan content was determined, however, the NCS + Zn-coated samples showed lower durability against washing, which is most pronounced in the case of TEMPO-oxidized and TOCN-coated viscose fabrics. The latter can be explained by reduced irreversible binding of NCS + Zn and viscose since in the case of NCS + Zn, unlike NCS, a certain number of amino groups of chitosan are unavailable for irreversible binding with viscose due to chelation with zinc ions.

Decrease in chitosan content, during washing, is followed by a simultaneous decrease in zinc content of viscose fabrics functionalized with NCS + Zn. The highest decrease in zinc content was recorded after the first washing cycle: 90% for CV/NCS + Zn, 90% for TEMPO CV/NCS + Zn, and 85% for TOCN CV/NCS + Zn. Further decrease in the zinc content was recorded after the third washing cycle: 82% for CV/NCS, 89% for TEMPO CV/NCS, and 88% for TOCN CV/NCS, as well as the fifth washing cycle: 41% for CV/NCS, 17% for TEMPO CV/NCS, and 24% for TOCN CV/NCS. The difference of total decrease in zinc and chitosan content after five washing cycles for all NCS + Zn functionalized fabrics: CV/NCS + Zn (99% and 49%, respectively), TEMPO CV/NCS + Zn (99% and 96%, respectively), and TOCN CV/NCS + Zn (99% and 87%, respectively) confirmed that a certain amount of free zinc ions, present in NCS + Zn dispersion, was bound directly to the fabrics during functionalization procedure. The discussed decrease in chitosan and zinc content in functionalized fabrics can be explained by a certain amount of NCS/NCS + Zn and zinc ions reversibly bound onto the fabric surface.

**Figure 7:**
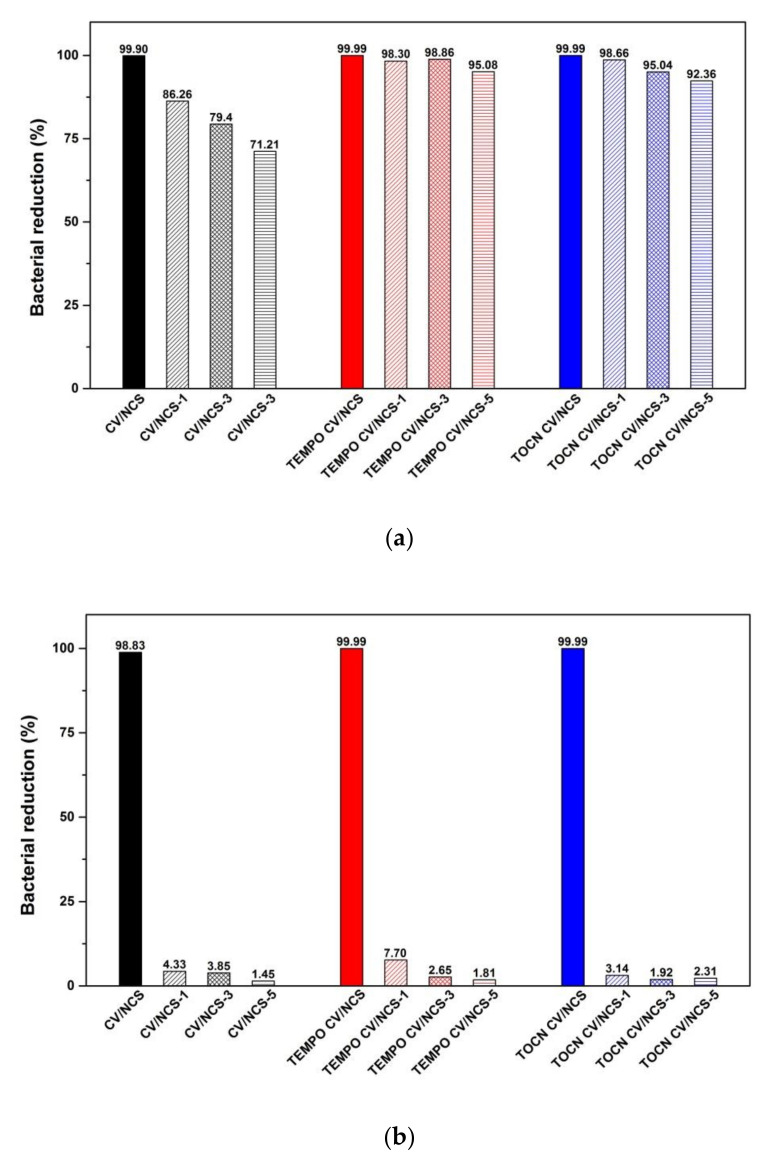

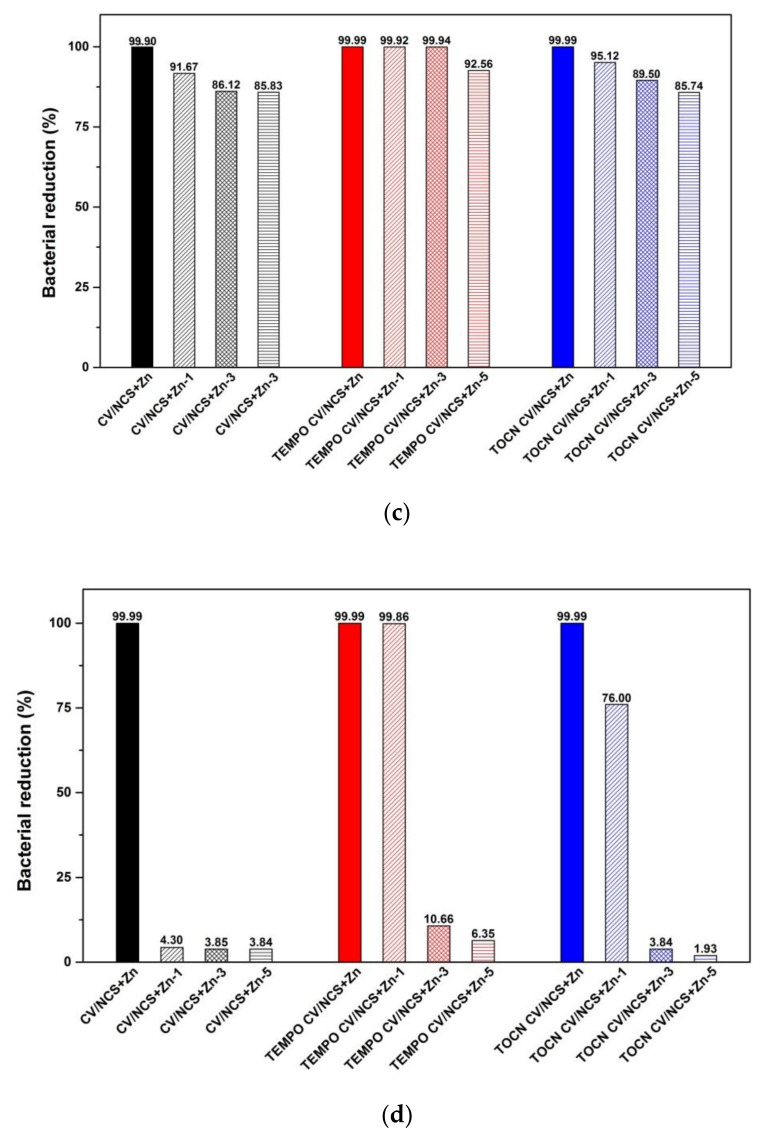
Bacterial reduction of NCS (**a**,**b**) and NCS + Zn (**c**,**d**) functionalized viscose fabrics after 1, 3, and 5 washing cycles against *S. aureus* (**a**,**c**) and *E. coli* (**b**,**d**); modified from [39].

The achieved antibacterial activity against *S. aureus* was durable for NCS functionalized pristine viscose up to three washing cycles, and even up to five washing cycles for NCS functionalized pretreated viscose fabrics (Figure 7a). On the other hand, for all NCS functionalized viscose fabrics, the antibacterial activity against *E. coli* was present only before washing (Figure 7b). Compared with the NCS functionalized, NCS + Zn functionalized pristine viscose fabrics showed more washing durable antibacterial activity against *S. aureus* (i.e., antibacterial activity was confirmed after five washing cycles, Figure 7c), while in the case of NCS + Zn functionalized pretreated viscose fabrics washing durable antibacterial activity against *E. coli* after only one washing cycle was achieved (Figure 7d). Therefore, the antibacterial activity against *S. aureus* is more durable than that against *E. coli*. However, such behavior cannot be clearly explained. Comparing the results obtained for pristine and pretreated viscose fabrics functionalized either with NCS or NCS + Zn, it is evident that pretreated fabrics have more effective and durable antibacterial activity against both types of bacteria whereby TEMPO oxidation is the most successful pretreatment method. A clear correlation between chitosan content and bacterial reduction of NCS and NCS + Zn functionalized viscose fabrics after washing was not observed, which can be explained by the fact that the bacterial reduction of chitosan functionalized materials does not only depend on chitosan content but also on the accessibility of chitosan’s amino groups. Only when chitosan’s amino groups are accessible and protonated, i.e., do not participate in bonding with other chitosan molecules, other components, or functionalized materials, can electrostatic interactions between them and the bacterial cell wall occur, leading to the bacterial reduction [38]. During washing, the decrease in chitosan content probably simultaneously occurs with changes in the accessibility of the chitosan’s amino groups due to reorganization of the bonds between them and other chitosan molecules, zinc ions, and viscose fabrics, resulting in an unclear correlation between chitosan content and bacterial reduction after washing.

## 4. Conclusions

In this study, antibacterial properties of viscose fabrics functionalized with chitosan nanoparticles (without and with embedded zinc ions) were improved by successful irreversible binding of antibacterial agents using two different pretreatments of viscose fabric: oxidation with TEMPO and coating with TOCN which provided carboxyl and aldehyde groups onto/into the viscose fabric. Both pretreatments resulted in more efficient and washing durable antibacterial activity of viscose fabrics regardless of the used antimicrobial agent. NCS functionalized pretreated viscose fabrics preserved antibacterial activity against *S. aureus* after five washing cycles, while they lost antibacterial activity against *E. coli* after one washing cycle. The addition of zinc ions into/onto the NCS led to an increased positive charge density of NCS and, consequently, more intense electrostatic interactions between NCS + Zn functionalized pretreated viscose fabrics and the bacterial cell wall: NCS + Zn functionalized pretreated viscose fabrics preserved antibacterial activity against *Escherichia coli* after one washing cycle. Presented results are promising in the area of textiles as high value-added products, which may find application as washable antibacterial medical textiles, especially for manufacturing clothes intended for people with decubitus or sensitive skin prone to wounds.

## Figures and Tables

**Table 1:** Denotation of the viscose fabric samples before and after washing *.

Sample Description	Number of Washing Cycles			
	0	1	3	5
Pristine viscose	CV	-	-	-
TEMPO-oxidized viscose	TEMPO CV	-	-	-
Viscose coated with TOCN	TOCN CV	-	-	-
CV functionalized with NCS	CV/NCS	CV/NCS-1	CV/NCS-3	CV/NCS-5
TEMPO CV functionalized with NCS	TEMPO CV/NCS	TEMPO CV/NCS-1	TEMPO CV/NCS-3	TEMPO CV/NCS-5
TOCN CV functionalized with NCS	TOCN CV/NCS	TOCN CV/NCS-1	TOCN CV/NCS-3	TOCN CV/NCS-5
CV functionalized with NCS + Zn	CV/NCS + Zn	CV/NCS + Zn-1	CV/NCS + Zn-3	CV/NCS + Zn-5
TEMPO CV functionalized with NCS + Zn	TEMPO CV/NCS + Zn	TEMPO CV/NCS + Zn-1	TEMPO CV/NCS + Zn-3	TEMPO CV/NCS + Zn-5
TOCN CV functionalized with NCS + Zn	TOCN CV/NCS + Zn	TOCN CV/NCS + Zn-1	TOCN CV/NCS + Zn-3	TOCN CV/NCS + Zn-5

* This table is part of Table 7 published in [39]. Copyright by Matea Korica.

**Table 2:** Chemical composition of the pristine and pre-treated viscose fabrics before and after NCS + Zn functionalization determined by XPS.

Samples	C (%at)	N (%at)	O (%at)	Si (%at)	Cl (%at)	P (%at)	Zn (%at)	S (%at)	Na (%at)
CV	75.3	1.2	20.7	2.7					
TEMPO CV	58.8	0.9	38.8	0.4	0.1				1.0
TOCN CV	64.9	1.0	31.5	2.6					
CV/NCS + Zn	68.0	3.6	24.7	1.5	0.4	0.7	1.2		
TEMPO CV/NCS + Zn	58.5	7.0	30.2	1.0	1.1	0.0	1.7	0.4	
TOCN CV/NCS + Zn	65.7	3.8	26.3	1.3	0.6	0.8	1.3	0.3	

**Table 3:** The chitosan and zinc content in the NCS and NCS + Zn functionalized viscose fabrics *.

Sample	CS, %	Zn, %
CV/NCS	0.43	
TEMPO CV/NCS	1.18	
TOCN CV/NCS	0.63	
CV/NCS + Zn	0.63	1.86
TEMPO CV/NCS + Zn	1.04	3.40
TOCN CV/NCS + Zn	1.00	2.05

* This table is modified Table 19 published in [39]. Copyright by Matea Korica.

**Table 4:** The bacterial reduction of NCS and NCS + Zn functionalized viscose fabrics *.

Sample	Bacterial Reduction, %	
	*S. aureus*	*E. coli*
CV/NCS	99.9	98.83
TEMPO CV/NCS	99.9	99.9
TOCN CV/NCS	99.9	99.9
CV/NCS + Zn	99.9	99.9
TEMPO CV/NCS + Zn	99.9	99.9
TOCN CV/NCS + Zn	99.9	99.9

* Results previously published in [39].

## Data Availability

The data presented in this study are available on request from the corresponding author.

## Supplementary Materials

The following are available online at https://www.mdpi.com/article/10.3390/ma14133762/s1: Figure S1, XPS survey spectra of pristine and pre-treated viscose fabrics before and after functionalization with NCS + Zn; Figure S2, Zeta potential of NCS and NCS + Zn; and Figure S3, Changes in isoelectric points with desorption of chitosan and zinc ions from viscose fabrics functionalized with NCS (a) and NCS + Zn (b,c) before and after 1, 3 and 5 washing cycles.
