# Supplementary material for: Chitosan Nanoparticles Functionalized Viscose Fabrics as Potentially Durable Antibacterial Medical Textiles
Source: Materials (Basel). 2021 Jul 5;14(13):3762. doi: 10.3390/ma14133762 (PMC8269808; doi:10.3390/ma14133762)
Supplement: Supplementary file 1 [file materials-14-03762-s001.zip › materials-1266172-supplementary.pdf]

materials

MDPI

Supplementary Materials

Chitosan Nanoparticles Functionalized Viscose Fabrics as Potentially Durable Antibacterial Medical Textiles

Matea Korica [1], Zdenka Peršin [2], Lidija Fras Zemljič [2], Katarina Mihajlovski [3], Biljana Dojčinović [4], Snežana Trifunović [5], Alenka Vesel [6], Tanja Nikolić [3], Mirjana M. Kostić [3]

[1] Innovation Center of Faculty of Technology and Metallurgy, University of Belgrade, 11000 Belgrade, Serbia; mkorica@tmf.bg.ac.rs
[2] Institute of Engineering Materials and Design, Faculty of Mechanical Engineering, University of Maribor, 2000 Maribor, Slovenia; zdenka.persin@um.si (Z.P.); lidija.fras@um.si (L.F.Z.)
[3] Faculty of Technology and Metallurgy, University of Belgrade, 11000 Belgrade, Serbia; kmihajlovski@tmf.bg.ac.rs (K.M.); tanjanikol@gmail.com (T.N.)
[4] Institute of Chemistry, Technology and Metallurgy, University of Belgrade, 11000 Belgrade, Serbia; bmatic@chem.bg.ac.rs
[5] Faculty of Chemistry, University of Belgrade, 11000 Belgrade, Serbia; snezanat@chem.bg.ac.rs
[6] Department of Surface Engineering, Jožef Stefan Institute, University of Ljubljana, 1000 Ljubljana, Slovenia; alenka.vesel@guest.arnes.si
* Correspondence: kostic@tmf.bg.ac.rs; Tel.: + 381-11-3303-628

Citation: Korica, M.; Peršin, Z.; Fras Zemljič, L.; Mihajlovski, K.; Dojčinović, B.; Trifunović, S.; Vesel, A.; Nikolić, T.; Kostić, M.M. Chitosan Nanoparticles Functionalized Viscose Fabrics as Potentially Durable Antibacterial Medical Textiles. *Materials* **2021**, *14*, 3762. https://doi.org/10.3390/ma14133762

Academic Editor: Alina Sionkowska

Received: 2 June 2021
Accepted: 3 July 2021
Published: 5 July 2021

Publisher's Note: MDPI stays neutral with regard to jurisdictional claims in published maps and institutional affiliations.

Copyright: © 2021 by the authors. Licensee MDPI, Basel, Switzerland. This article is an open access article distributed under the terms and conditions of the Creative Commons Attribution (CC BY) license (http://creativecommons.org/licenses/by/4.0/).

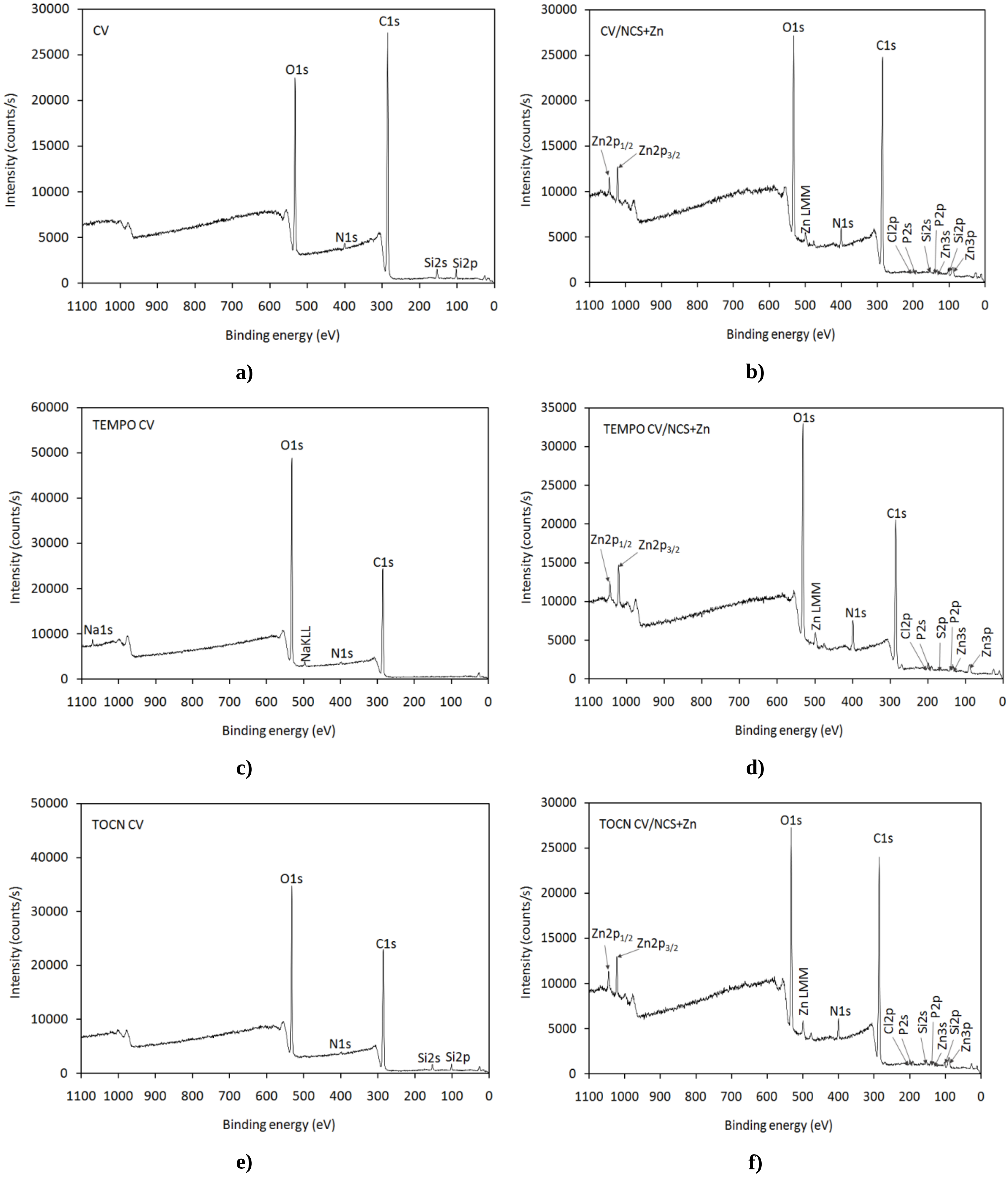

Figure S1. XPS survey spectra of pristine and pre-treated viscose fabrics before and after functionalization with NCS+Zn.

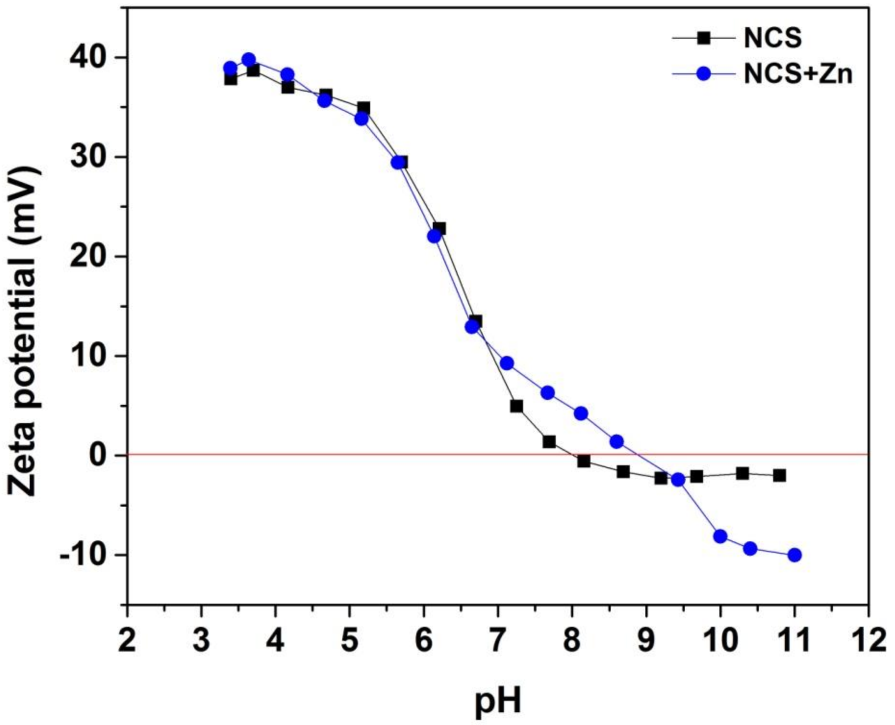

Figure S2. Zeta potential of NCS and NCS+Zn

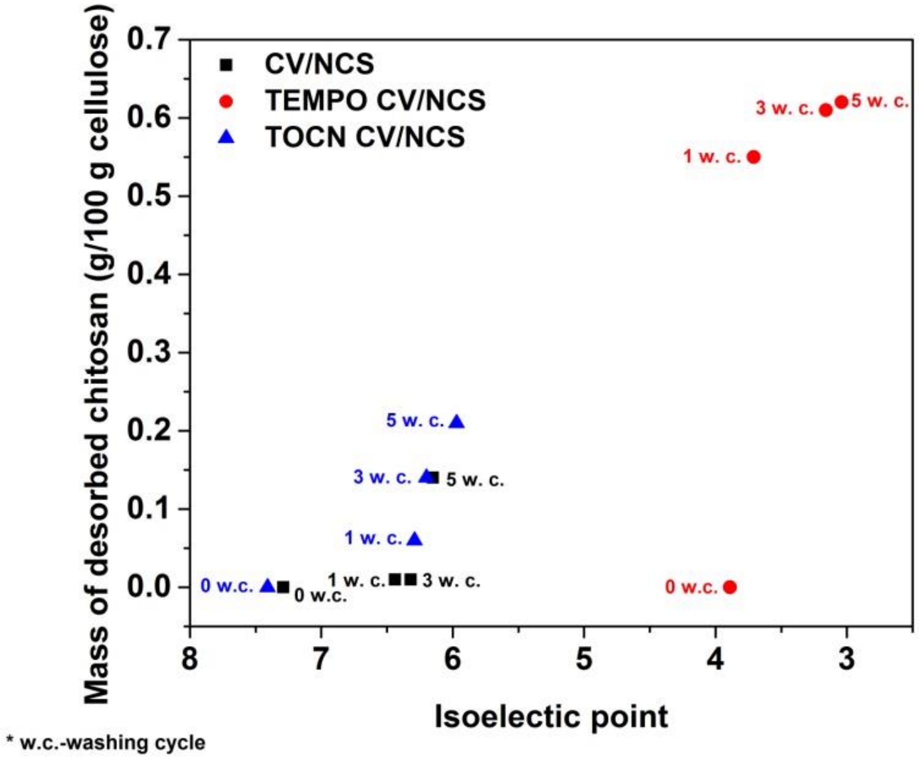

a)

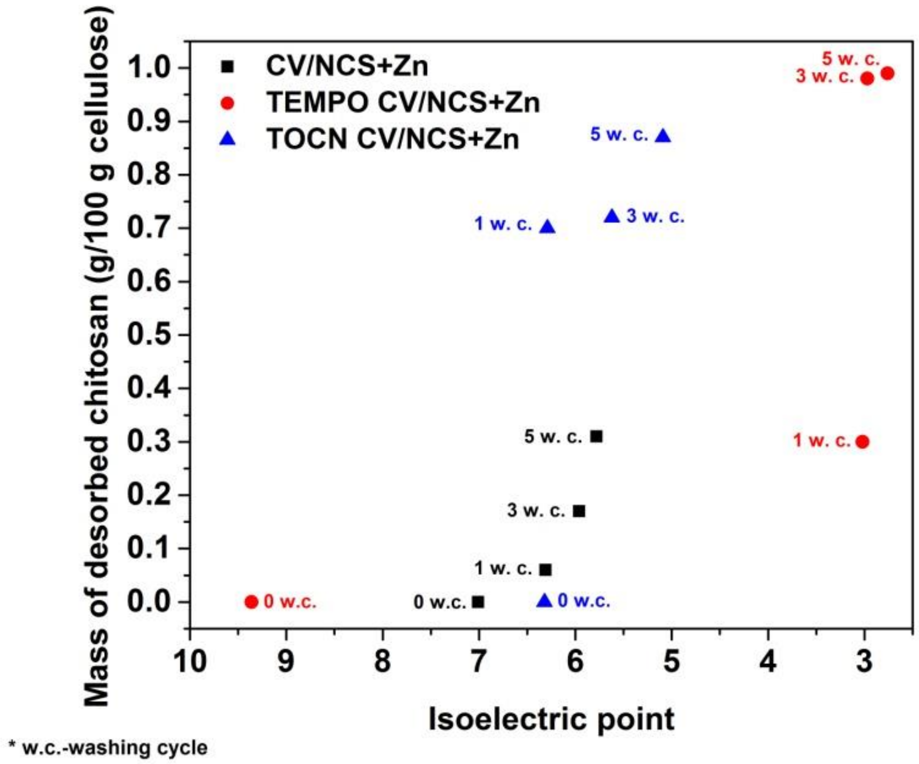

b)

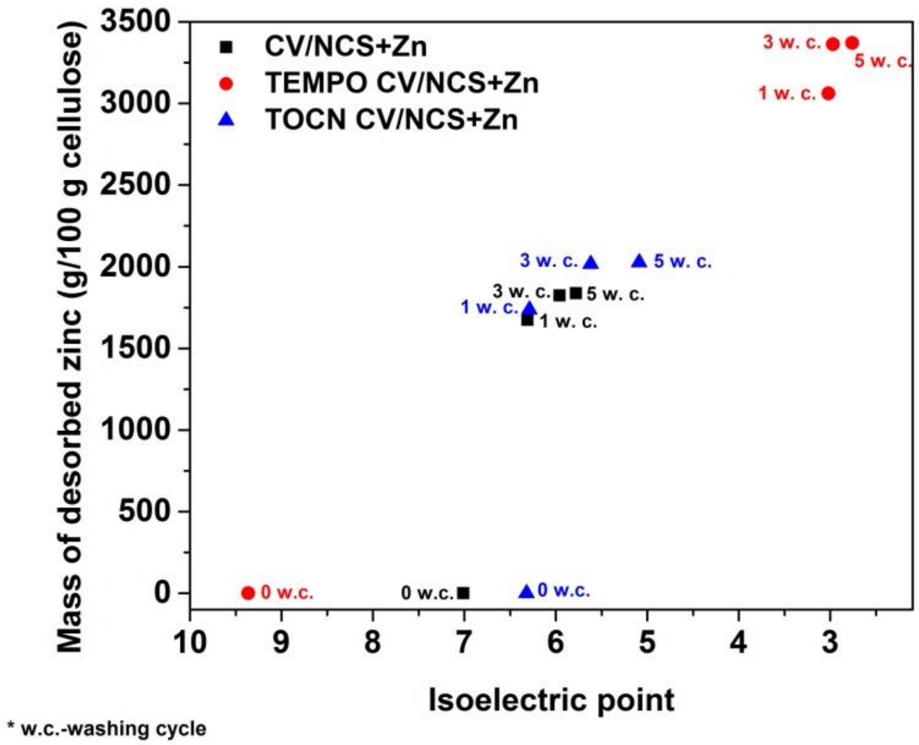

c)

Figure S3. Changes in isoelectric points with desroption of chitosan and zinc ions from viscose fabrics functionalized with NCS (**a**) and NCS+Zn (**b, c**) before and after 1, 3 and 5 washing cycles.
